# Integrating Community-Based Recovery Models into Systems of Care: Creating Recovery-Oriented Environments

**DOI:** 10.1192/j.eurpsy.2026.11658

**Published:** 2026-07-31

**Authors:** E. Adamberry Olivero, on behalf of Clubhouse Europe, A. E. Segovia Martin

**Affiliations:** Clubhouse Europe, Gibraltar, Gibraltar

## Abstract

**Introduction:**

Modern mental health systems increasingly recognize that recovery extends beyond clinical stabilization. To achieve sustainable outcomes, care must integrate medical, social, and community dimensions that empower individuals to rebuild meaningful lives. The Clubhouse Model—originating at Fountain House in 1948—demonstrates how community-based programs can bridge this gap by creating structured, recovery-oriented environments that complement clinical services and address the social determinants of health.

**Objectives:**

This presentation calls for the inclusion of community-based models, such as Clubhouses, within national and regional systems of mental health care. It aims to highlight how recovery-oriented environments strengthen integrated care frameworks by fostering participation, belonging, and sustained recovery beyond treatment settings

**Methods:**

Drawing from international evidence and longitudinal studies, this analysis reviews the Clubhouse Model’s outcomes in employment, social inclusion, and health stabilization. It also explores collaborative approaches between Clubhouses and healthcare providers that promote continuity, accessibility, and holistic support across care systems.

**Results:**

Research demonstrates that Clubhouses:

- Reduce hospitalizations and emergency care utilization.
- Improve employment and education outcomes.
- Enhance empowerment, social inclusion, and recovery.

Lower long-term healthcare costs by supporting prevention

**Conclusions:**

To create truly recovery-oriented systems, healthcare must extend beyond the clinic to include environments that sustain connection, meaning, and community participation. Integrating Clubhouses and similar community-based programs within national systems of care offers a practical, evidence-based path toward more inclusive, effective, and humane mental health recovery.

**Disclosure of Interest:**

None Declared

